# Inkjet Printing of Magnetic Particles Toward Anisotropic Magnetic Properties

**DOI:** 10.1038/s41598-019-52699-0

**Published:** 2019-11-07

**Authors:** Karam Nashwan Al-Milaji, Ravi L. Hadimani, Shalabh Gupta, Vitalij K. Pecharsky, Hong Zhao

**Affiliations:** 1 Virginia Commonwealth University, Department of Mechanical and Nuclear Engineering, BioTech One, 800 East Leigh Street, Richmond, VA 23219 USA; 2 Virginia Commonwealth University, Department of Mechanical and Nuclear Engineering, 401 West Main Street, Richmond, VA 23284 USA; 3 Ames Laboratory of the U.S. Department of Energy, Division of Materials Science and Engineering, Iowa State University, Ames, IA, 50011-2416, USA; 4 Department of Materials Science and Engineering, Iowa State University, Ames, IA, 50011-1096, USA

**Keywords:** Applied physics, Chemical engineering, Mechanical engineering

## Abstract

Unique properties of one-dimensional assemblies of particles have attracted great attention during the past decades, particularly with respect to the potential for anisotropic magnetism. Patterned films can be created using inkjet printing; however, drying of particle-laden colloidal droplets on solid surfaces is usually accompanied by the well-known coffee-ring effect, deteriorating both the uniformity and resolution of the printed configurations. This study examines the effect of externally applied magnetic field on particle deposition patterns. Ferromagnetic Gd_5_Si_4_ particles were formulated in terpineol oil and directly deposited via magnetic field-assisted inkjet printing on a photopaper to generate patterned films with suppressed coffee-ring effect. The particle deposition morphology is determined by both solvent imbibition and particle-magnetic field interactions. Three characteristic times are considered, namely, the critical time for solvent imbibition into the substrate (*t*_*im*_), the time it takes for particles to form chains in the presence of the magnetic field (*t*_*ch*_), and the time in which the particles reach the substrate in the direction normal to the substrate (*t*_*pz*_). The characteristic time ratios (*t*_*pz*_/*t*_*im*_) and (*t*_*pz*_/*t*_*ch*_) determine the final deposition morphology in the presence of magnetic field. The ability to control particle deposition and assembly, thus tuning the magnetic anisotropic properties of nanostructured materials is a promising approach for many engineering applications.

## Introduction

One-dimensional (1D) chain structures have attracted tremendous attention during the past decades owing to unique shape anisotropies and magnetic characteristics^1^. The major benefit of 1D particle assemblies is the ability to tailor magnetic, mechanical, electrical, and optical properties^2^. Such particle assemblies are of tremendous interest in many technological applications such as high density data storage systems^3^, magnetic sensors^4^, and magnetic refrigerators^5^, to name a few.

The chaining, or the formation of the so-called magnetosomes, is a result of intrinsic magnetic anisotropy, where magnetic particles orient and assemble with their easy magnetization axes (magnetization vectors) parallel to the magnetic field vector to minimize the magnetostatic energy of individual nanoparticles^6,7^. This phenomenon combines the magnetic moments present in every magnetic particle in a particular direction, which is considered a promising venue for fabricating functional devices^8,9^.

One approach that has a great potential in designing and fabricating magnetic devices is inkjet printing. Unlike a number of thin-film deposition and photolithography techniques, inkjet printing is a versatile and cost-effective tool by which the alignment and chaining of magnetic particles could be well-controlled^10^. However, the well-known coffee-ring effect (CRE) is a known impediment present in inkjet printing applications, and it should be addressed before broad adoption of this approach^11–13^. The CRE phenomenon has been the subject of broad investigations and scrutiny to unveil the underlying mechanisms^14,15^. Under certain conditions, when a particle-laden droplet is left to dry on a nonporous substrate, the colloidal particles transport to the three-phase contact line (TCL) resulting in a ring-like particle deposition^16,17^. Two decades ago, Deegan *et al*. attributed the cause of CRE to non-uniform evaporation rate across the air-liquid interface, which generates a lateral convective flow responsible for the particle migration toward the TCL^18–20^. To date, most of the research dedicated to mitigate or suppress the CRE was focused on conventional means, such as controlling the solvent drying conditions^21–23^, modifying particle shapes^24,25^, tuning solvent properties^26^, adjusting substrate wettability^27,28^, and reversing the lateral-convective flow through introducing Marangoni flow^29–32^. However, the effects of porous substrates and external magnetic field on the CRE when the colloidal particles are magnetic require further investigations.

The main objective of this study is to elucidate the effect of external magnetic field on the magnetic particle assembly during inkjet printing to obtain anisotropic magnetic patterns through particle chaining with suppressed CRE formation. Ferromagnetic Gd_5_Si_4_ particles were formulated in terpineol oil and printed onto a porous substrate to form patterned films. A porous substrate (photopaper) used in this study quickly removes the solvent through imbibition, which suppresses the CRE to a certain degree. When no magnetic field is applied, patterns ranging from uniform deposits to clear rings have been produced depending on the droplet size and particle loading. With the magnetic field, however, the CRE can be suppressed or significantly reduced, and the magnetic nanoparticles assemble into chains, producing anisotropic magnetic patterns. Three characteristic times, namely the critical time for solvent imbibition into the substrate (*t*_*im*_), the time for particles to chain along the direction of the magnetic field (*t*_*ch*_), and the time for the particles to reach the substrate in the direction normal to the substrate (*t*_*pz*_) under the magnetophoretic force, are considered in this work. These characteristic times demonstrate the competition between particle chaining and deposition in the presence of magnetic field versus solvent imbibition, which are directly related to the morphology of the particle deposition. Such ability to control the magnetic particle deposition and assembly through inkjet printing provides a great potential for many engineering and technological applications.

## Results and Discussion

Particle-laden droplets were jetted on a photopaper substrate, where the solvent removal through infiltration and evaporation occur simultaneously. However, for terpineol oil, the infiltration occurs at a much faster rate than evaporation. In this study, the size of the printed droplets was controlled by jetting multiple bursts of colloidal droplets, where three-phase contact lines remain pinned during the solvent imbibition as shown in the Supporting Information (Fig. S1). We note that TCL pinning on solid substrates is usually determined by the substrate wetting property, which is mainly dependent on surface roughness^33,34^ and chemical homogeneity^35–37^. The initial pinning of solvents on substrates could be further enhanced by introducing suspended particles to anchor the contact line^18,38^. The TCL pinning of terpineol oil loaded with ferromagnetic particles on a porous substrate could be attributed to fast solvent imbibition, surface roughness of the photopaper, and low surface tension and high viscosity of the oil. Generally, non-uniform evaporation flux across the curved interface of sessile droplets initiates a radial evaporation-induced flow that drives the particles to the TCL. This is usually combined with particle diffusion in the bulk of the deposited droplets. Yet, the low vapor pressure and high viscosity of terpineol oil results in extremely low radial and diffusion velocities of the particles (Fig. S2). Therefore, it is reasonable to neglect the evaporation of terpineol oil and the associated radial particle transport and particle diffusion in the following discussion.

**Figure 1:**
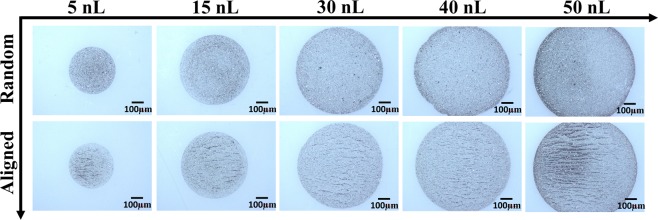
Optical images of particle depositions of different droplet volumes, printed with 25 mg/mL particle concentration. The top row designated as “Random” illustrates samples printed without external magnetic field. The bottom row designated as “Aligned” illustrates samples with one-dimensional chains of particles formed during printing in the presence of applied magnetic field.

Without applied magnetic field, various particle deposition patterns were observed depending on the droplet volume. Different from conventional CRE, where colloidal particles transport to the edge of sessile droplets by virtue of the evaporation-induced capillary flow, in this study, solvent imbibition is the main driving mechanism that dictates the particle deposition pattern when no magnetic field is applied. Solvent in small droplets can be fully imbibed into the porous substrate directly underneath the droplets (primary imbibition stage), where the colloidal nanoparticles are immobilized and deposited onto the substrate due to fast solvent removal. For larger droplets, however, the solvent cannot be completely removed through primary imbibition, and its excess is removed through a much slower lateral imbibition (secondary imbibition stage) into the regions surrounding the as-deposited droplets. In this case, the colloidal particles can be driven to the TCL during the secondary imbibition process, producing deposits similar to conventional CRE. The images in the top row of Fig. 1 illustrate particle deposition patterns for various sizes of printed colloidal droplets with 25 mg/mL particle concentration when no magnetic field is applied. For droplet volumes of about 30 nL and smaller, primary imbibition is dominant and nearly uniform particle depositions without accumulation at the TCL are observed owing to fast solvent removal that prevents particle transport to the periphery of the jetted droplet. Particles begin to accumulate at the edges of sessile droplets with larger than 30 nL volumes, indicating that secondary imbibition stage plays a role, where many particles are now transported to the TCL as the remaining solvent is slowly removed laterally. Printing colloidal inks with a lower particle concentration of 10 mg/mL follows a similar trend in the pattern formation whereas the CRE becomes visible when droplet volumes reach about 40 nL (Fig. S3).

Results described in the previous paragraph can be compared with those reported by Duo and Derby who studied particle accumulation at edges of sessile droplets on various porous substrates, including photopapers and ceramic powder beds, but without examining the influence of droplet volume^39,40^. They showed that Marangoni flow leads to suppression of the CRE due to the change in solvent composition during evaporation when depositing the droplets on a solid non-porous substrate. In case of photopapers and ceramic powder beds, Marangoni flow is reduced inside sessile droplets since solvent composition does not change during imbibition, leading to ring formation. The effects of different droplet and particle sizes, as well as pore diameters on the ring formation were investigated by Pack and coworkers^41^. In their study, the porosity of the substrate was limited to only vertical pores that were normal to the substrate surface, allowing only primary solvent imbibition without secondary (lateral) imbibition. Therefore, when primary imbibition terminates, ring formation is controlled by the evaporation of the remaining solvent. For substrates with large pore sizes solvent could be completely and quickly removed, suppressing CRE.

The mechanism of solvent removal in our study is different since the terpineol oil is nearly exclusively removed by infiltration into the porous substrate rather than through evaporation. As follows from our experiments, a 30 nL sessile droplet takes about 98 s to be completely drained, whereas a 50 nL droplet drains within approximately 143 s. The former can be considered as the critical characteristic imbibition time for ring formation, since the particle accumulation at the contact line is observed for droplet volumes starting from 30 nL. Fig. S4 and Video S1 in the Supporting Information illustrate the process of solvent drainage into the photopaper.

The location on which the sessile droplet is deposited has a limited capacity of solvent absorption. Once the jetted droplet impacts the photopaper, it loses approximately 11% of its entire volume within 500 ms by imbibition. Then, the solvent further seeps in at a slower rate into the porous substrate isotropically in all directions. When the deposition area underneath the droplet becomes saturated with terpineol oil, the solvent can only infiltrate laterally beyond the original footprint of the droplet. We note that the contact line of the droplet is pinned on the substrate and solvent seepage refers to the solvent infiltrated into the substrate. This, in turn, generates a radial flow that drives some of the particles toward TCL. However, this is not a concern for small sessile droplets (i.e., droplet volumes smaller than 30 nL), where the solvent is completely absorbed by the photopaper before noticeable particle transport to the contact line, resulting in a uniform particle deposition through immobilizing of the colloidal particles (Fig. 1). For larger droplets, the solvent eventually generates a larger footprint underneath the droplet as it continues to seep through the photopaper, resulting in deposition of magnetic particles on the photopaper, surrounded by a ring of terpineol oil within the substrate (Video S1). It is worth noting that the contact line of the droplet remains pinned at the original TCL during the solvent imbibition process (Fig. S1). Nilghaz *et al*. reported a similar observation in an effort to study dye stain formation on paper substrates, that is the solvent wicking front propagates further into the porous substrate than the dye where the dye molecules were separated from and left behind the water solvent^42^. They attributed this behavior to the capillary penetration into the paper, where the dye molecules were preferentially retained by the paper fibers. In our study, the colloidal particles are much larger than the pore size of the photopaper, therefore, the particle penetration is excluded. Instead, the particles deposit on the surface of the photopaper, forming different particle deposition patterns depending on the droplet volume.

**Figure 2:**
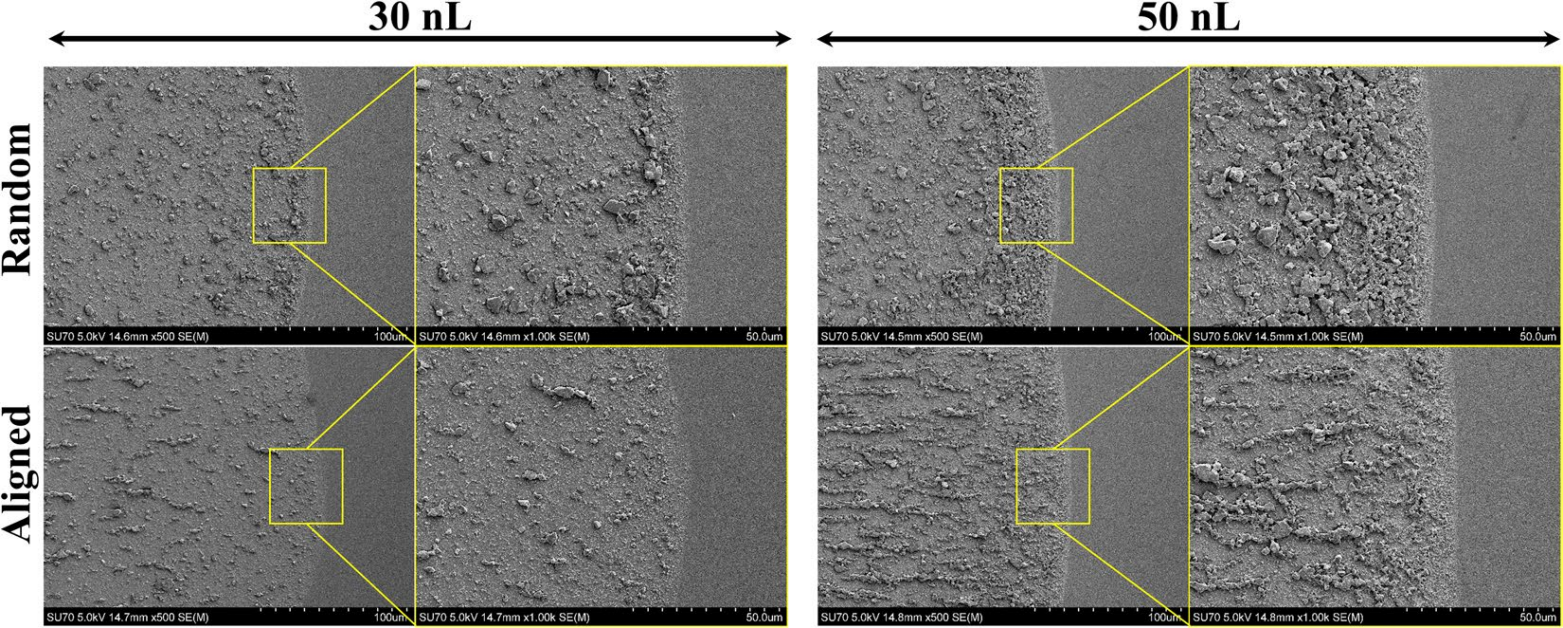
Scanning electron microscope (SEM) images demonstrating the difference in particle accumulation at the TCL region with and without applying magnetic field for droplet volumes of 30 nL and 50 nL. The images present a clear evidence of the reduced number of particles deposited at the TCL when the magnetic field was applied. The particle concentration was 25 mg/mL.

**Figure 3:**
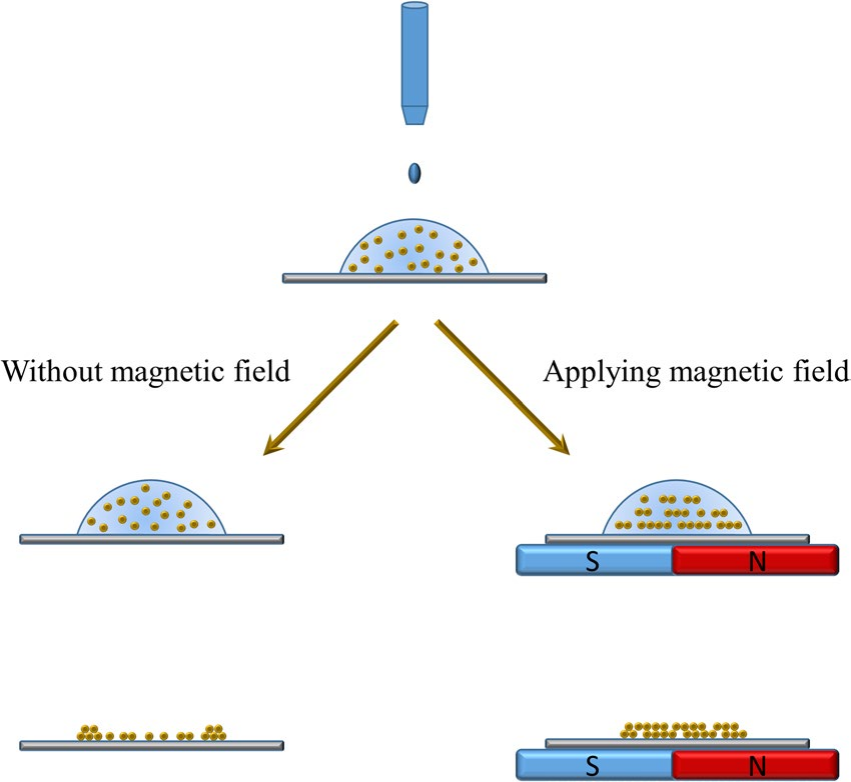
Schematic illustration of the magnetic field effect on ferromagnetic particle deposition. Ring-like depositions are produced when no magnetic field is applied, whereas, aligned chain-like patterns with much suppressed CRE are formed when a magnetic field is applied.

The application of an external magnetic field significantly impacts the colloidal ferromagnetic particle deposition in sessile droplets^43^. Intrinsic magnetocrystalline anisotropy of Gd_5_Si_4_ magnetic particles results in strong dipole interactions among the particles. These particles are forced to chain and assemble with their easy magnetization directions along the direction of the external magnetic field vector. The horizontal component of the magnetic field drives the magnetic particles to form long chains, especially for larger droplets. In addition, applying magnetic field significantly suppresses the ring formation. The underlying mechanism of suppressing the CRE by exerting magnetic field is that, the magnetic particles transport toward the substrate under the magnetophoretic force, represented by the magnetic field gradient in a direction perpendicular to the substrate. This inhibits the radial particle transport and deposition at the TCL due to solvent imbibition, especially for larger droplets. In addition, the colloidal particles are arrested by forming chains instead of being dragged to the contact line region by the lateral solvent flow during the secondary solvent imbibition stage. This, in turn, also contributes to the suppression of the CRE by limiting the number of individual particles that can migrate toward the contact line. Figures 1 and 2 display the chain formation and suppression of the coffee ring effect when printing droplets with colloidal magnetic particles under the influence of magnetic field. The ring formation has been significantly suppressed especially for the 50 nL droplets. Figure 3 is a schematic illustrating the difference between particle deposition patterns with and without the presence of magnetic field.

**Figure 4:**
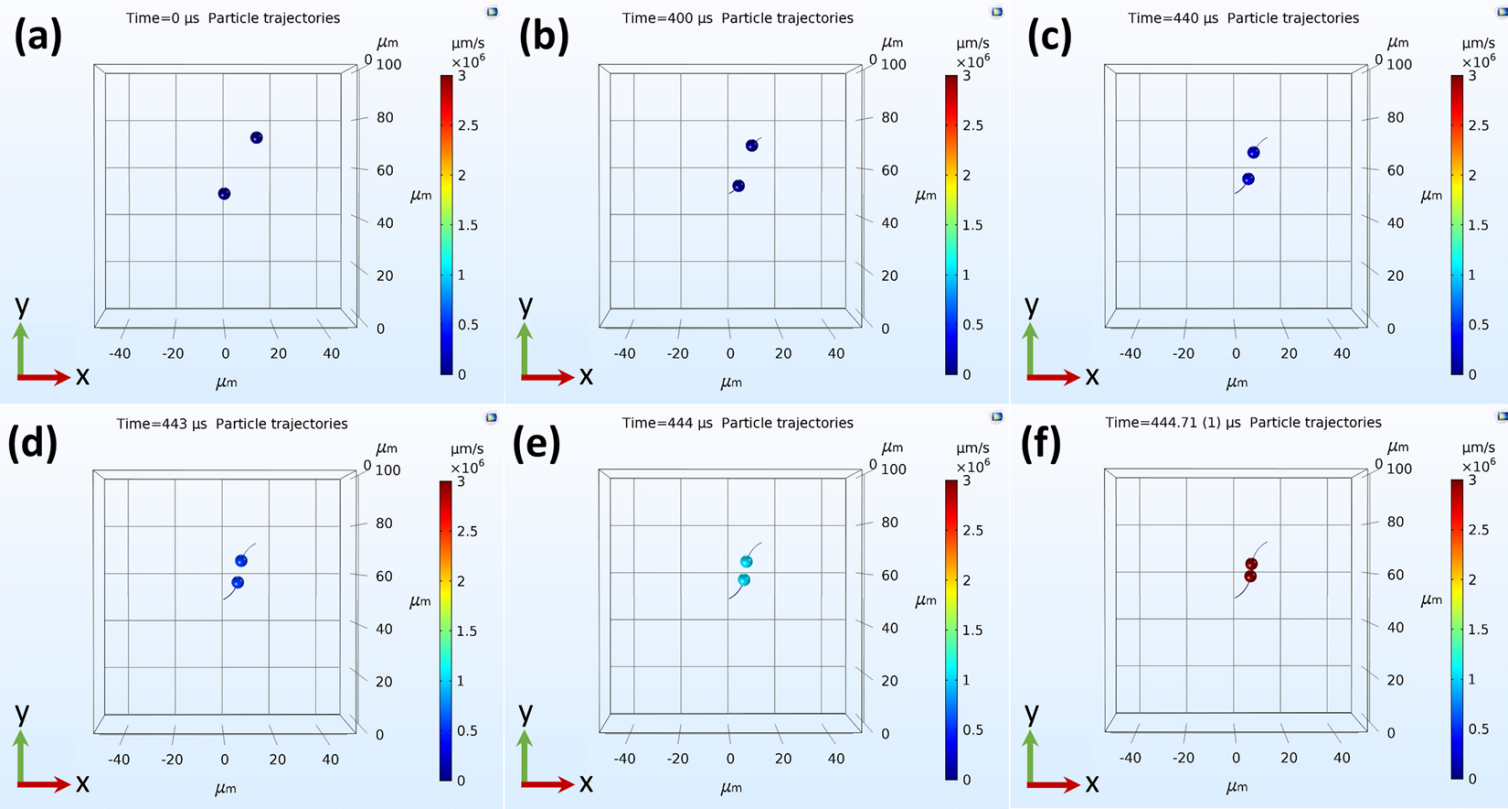
(**a**–**f**) Demonstration of chain initiation process by two magnetic dipoles in the presence of magnetic field. The dipoles are initially positioned with an orientation angle *α* = 30° with respect to the magnetic field vector. The magnetic field vector direction coincides with the positive y-axis. The elapsed time for the two dipoles to collide and initiate the chain is 446 µs. In this case, the dipoles travel in elliptical paths until they align and collide.

In order to better understand the influence of applying external magnetic field on ferromagnetic particle deposition, COMSOL Multiphysics was employed to simulate the particle chaining and deposition processes. To simulate the particle chaining, only two magnetic dipoles were introduced to the simulation domain, where the interaction force between the two dipoles is significantly affected by their initial orientation angle (*α*) with respect to the external magnetic field (Fig. S5). Figure 4 and Video S2 demonstrate the chaining process by two dipoles, one of which is placed at the center of the domain. The second dipole is positioned at 30° angle with the direction of the magnetic field (the positive direction of the y-axis coincides with the positive direction of the magnetic field). The initial distance between the two diploes corresponds to the mean particle distance (*L*_*m*_) calculated for 25 mg/mL particle concentration. Details about the modeling are provided in the Methods section. Videos S2–S6 display the chaining process with dipoles positioned at different orientation angles with the magnetic field vector, such as 0°, 30°, 45°, 60°, and 90°. Figure S6 shows the time elapsed for the dipoles to collide and chain (*t*_*ch*_). Figure S7 shows the interaction forces exerted on each particle at different initial orientation angles, which determines whether they approach or move away from each other. When both dipoles are placed along the x-axis (*α* = 90°), the force is repulsive and the dipoles move apart. On the contrary, the dipoles attract the most when *α* = 0° as shown in Video S3. In this case, the particle velocity continuously increases until the dipoles collide. For any other orientation (0° < α < 90°), the dipoles follow elliptical trajectories until they collide and align themselves with the y-axis, i.e., the direction of the magnetic field. The characteristic chaining time (*t*_*ch*_) increases with increasing *α*, which can be attributed to the competition between repulsion and attraction forces (Fig. S7).

The magnetophoretic force suppresses the CRE through attracting the magnetic particles toward the substrate. This force is generated by the magnetic gradient in the z-direction, which is perpendicular to the substrate since the permanent magnet is positioned underneath the substrate. The full magnetic field of the simulation domain is shown in the Supporting Information (Fig. S8a,b). Figure S8c shows the magnetic field gradient in the z-direction developed inside the droplet. In this domain, the particles are released at a distance (*L*_*m*_) away from the substrate in order to estimate the characteristic time (*t*_*pz*_) needed for magnetic particles to reach the substrate under the magnetophoretic force (Fig. S9). At the early stages of particle motion, the particle velocity starts to gradually increase, reaching the maximum of 108 µm/s in 238 ms, and then remains constant until the particles reach the substrate (Video S7).

The influence of exerting magnetic field on particle assembly and deposition can be determined by comparing the characteristic time needed for the particles to chain (*t*_*ch*_) and the time elapsed for particles to transport vertically toward the substrate (*t*_*pz*_) with the critical time for solvent imbibition (*t*_*im*_). The characteristic time ratios (*t*_*pz*_/*t*_*im*_) and (*t*_*pz*_/*t*_*ch*_) determine the final particle deposition and assembly in the presence of magnetic field. The characteristic time ratio (*t*_*pz*_/*t*_*im*_) determines whether the colloidal particles have sufficient time to reach the substrate before the solvent is completely drained. Rings form when *t*_*pz*_/*t*_*im*_ > 1, where the particle transport toward the substrate under the magnetophoretic force is much slower than the imbibition process, thus resembling no magnetic field condition. In this case, the particles follow the infiltration flow that persists during secondary imbibition stage, resulting in the ring formation. However, when *t*_*pz*_/*t*_*im*_ <1, the CRE is suppressed and the particles deposit on the substrate under the magnetophoretic force before the solvent is completely drained. On the other hand, the characteristic time ratio (*t*_*pz*_/*t*_*ch*_) exemplifies the competition between the particle chaining and particle deposition (immobilization) when the magnetic field is applied. For *t*_*pz*_/*t*_*ch*_ < 1, the magnetic particles deposit onto the substrate forming uniform but random depositions without obvious chain formation. In contrast, *t*_*pz*_/*t*_*ch*_ > 1, describes chain formation in the liquid phase before the particles are deposited on the substrate.

The experimental outcomes are corroborated by the analysis of characteristic time ratios when considering the critical solvent imbibition time for a 30 nL droplet. The corresponding characteristic time ratio *t*_*pz*_/*t*_*im*_ = 2.5 × 10^−3^ is orders of magnitude smaller than 1. This indicates suppression of the CRE, where the particles rapidly transport toward and deposit on the substrate before the solvent is completely infiltrated. In addition, the characteristic time ratios *t*_*pz*_/*t*_*ch*_ for the orientation angles of 0°, 30°, 45°, and 60° are 762.2, 560.5, 361.3, and 72.1, respectively. Hence, for all simulated cases except α = 90°, the particles chain in the liquid phase before depositing on the substrate. The combined effects of the magnetophoretic force and the chaining forces prompt the particles to form chains in the liquid phase along with the accelerated particle deposition onto the substrate. This explains the effect of magnetic field on the chain formation, as well as suppression of CRE, which is in a good agreement with our experimental observations.

### Implication in printed anisotropic magnetic films

**Figure 5:**
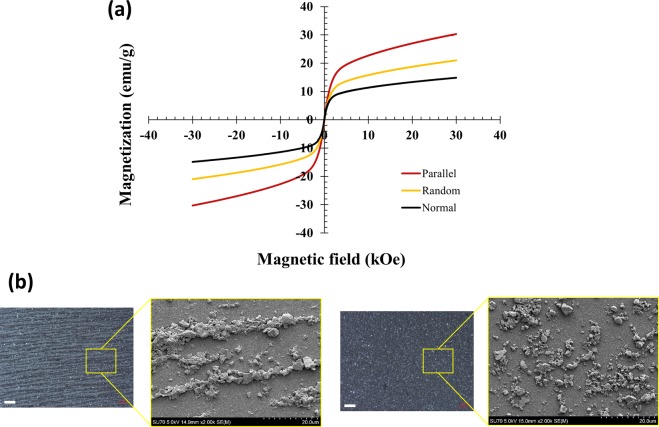
(**a**) Magnetization of printed films measured as a function of magnetic field. “Random” refers to films printed without external magnetic field. “Parallel” and “Normal” refer to films printed in magnetic field with magnetic particle chains, respectively, parallel and perpendicular to the measuring magnetic field vector. (**b**) Optical and SEM images of the films printed with (left) and without (right) magnetic field, illustrating chain formation in the films printed in the presence of external magnetic field. The scale bar in the optical images is 100 µm.

In order to evaluate magnetic properties of different patterns of magnetic particles, continuous films were generated by this inkjet printing process with and without external magnetic field. The printed films were characterized with a physical property measurement system (PPMS) and SEM. Figure 5a illustrates magnetization curves of the printed films measured at 300 K. The corresponding patterns formed by particles during printing with and without magnetic field are illustrated in Fig. 5b. Higher magnetization values were obtained when measuring films with 1D chains parallel to the direction of the measuring magnetic field, as compared to the magnetization values of the films with random particle orientation. This confirms that chains are formed by particles in nearly identical orientations, and that the chain axes coincide with the easy magnetization directions of the individual particles. Conversely, the lowest magnetization values were observed when the 1D chains were normal to the direction of the magnetic field. The difference in magnetization is due both to intrinsic magnetocrystalline anisotropy of Gd_5_Si_4_, and to shape anisotropy of 1D chains. Samples with random particle orientation (i.e., printed without applying magnetic field) were tested at different orientations with respect to direction of the measuring magnetic field vectors and are shown in Fig. S10. We also note that the photopaper employed in this study contributes weak but measurable magnetic background, as illustrated in the Supporting Information (Fig. S11). The background from the substrate was subtracted from the magnetization data illustrated in Figs. 5a and S10.

In summary, 1D chain structures of magnetic particles with suppressed ring formation have been obtained on porous substrates through the magnetic-field assisted inkjet printing process. Gd_5_Si_4_ magnetic particles were formulated in terpineol oil and printed on photopapers. We demonstrate that CRE can be suppressed and particle deposition morphology can be controlled by solvent imbibition, whereas particle chaining, and particle transport can be further tailored making use of magnetic interactions. When no magnetic field was applied during printing, different particle depositions were observed depending on the particle concentration and the volume of the jetted droplet. For small droplets, no ring formation was observed because the solvent can imbibe into the substrate in a short period of time (i.e., less than the critical imbibition time). However, the solvent imbibition time for large colloidal droplets exceeds the critical imbibition time mostly due to the second stage of solvent seepage. This, in turn, generates a radial flow that drags some of the colloidal particles to the periphery of the sessile droplet. Applying magnetic field enables the formation of 1D chain structure through assembling the magnetic particles along their easy axes. It also significantly suppresses the CRE through limiting particle transport to the TCL by means of particle chaining and magnetophoretic force, especially for larger droplets. Numerical simulations predicted that colloidal magnetic particles assemble in the liquid phase before they vertically transport and deposit on the substrate, which agrees very well with our experimental findings. Finally, films printed in the presence of magnetic field exhibit anisotropic magnetic response to the applied magnetic field due to the 1D assembly structure, which holds a great potential in many engineering applications.

## Methods

### Inkjet printing of magnetic particles

In this study, a photopaper (Kodak, 8.5 × 11″ Gloss) was cut into 20 mm × 20 mm and used as a porous substrate for the inkjet printing process. Gd_5_Si_4_ magnetic particles were prepared by ball milling at the Ames laboratory^44,45^. The particles were dispersed in terpineol oil (Alpha-Terpineol, 96% purity, Alfa Aesar) to obtain particle concentrations of 10 mg/mL and 25 mg/mL. In order to enhance the adhesion of the Gd_5_Si_4_ particles with the substrate, 0.15 wt% ethyl cellulose (18–22 mPa·s, 5% in toluene + ethanol (80:20) at 25 °C, TCI America) was added to the dispersion to form the magnetic ink. Then, the magnetic ink was ultrasonicated for 10 min before printing.

The magnetic ink with different particle concentrations was jetted on the photopaper substrate via an inkjet printing platform (Jetlab 4, MicroFab). A piezoelectric nozzle with an orifice size of 80 µm (MJ-ATP-01-80-8MX, MicroFab) was employed in this study, driven by a waveform generator (Jetdriver III, MicroFab). The nozzle was heated to 50 °C to facilitate the jetting process of terpineol oil. The size of the jetted droplets on the photopaper was controlled by the number of bursts, where the volume of individual jetted droplet was ~500 pL generated at 200 Hz jetting frequency. The alignment of the magnetic particles during printing was achieved by placing a neodymium permanent magnet (BC14-N52, K&J Magnetics) underneath the substrate to force the nanoparticles to align along the direction of the externally applied magnetic field. Samples with random particle orientations were printed without the permanent magnet.

### Morphology characterization

An ultra-high-resolution scanning electron microscope (HITACHI SU-70 FE-SEM) with 5 kV and 15 mm scanning distance was used for morphology characterization of the printed patterns. To minimize charging effect of the printed magnetic particles, the samples were coated with platinum using a platinum sputter (Denton Vacuum Desk V) for 120 s.

### Magnetic characterization

Magnetic properties of the printed patterns were characterized using a physical property measurement system (PPMS) from Quantum Design. The size of the printed films was adjusted to 4 mm × 4 mm, printed using ink with 25 mg/mL particle concentration. Isothermal magnetization was measured at T = 300 K in magnetic field varying between −30,000 Oe and +30,000 Oe with field sweep rate of 10 Oe/s. The total mass of the magnetic particles on the substrate was calculated based on the number and volume of droplets to form the film and the ink concentration. Each sample was measured with film surface parallel and normal to the direction of the measuring magnetic field vector.

### Numerical simulations of magnetic field and particle motion

Two simulation domains were constructed in COMSOL Multiphysics (version 5.4) to estimate the characteristic times *t*_*ch*_ and *t*_*pz*_. The first simulation domain couples the magnetic field and particle tracking with the fluid flow together to simulate the characteristic time (*t*_*pz*_) needed for the particles to migrate toward and reach the substrate from a distance *L*_*m*_ away the substrate surface. With the assumption that the colloidal magnetic particles are homogeneously dispersed in the bulk of the sessile droplet, the distance *L*_*m*_ is taken as the mean distance between two colloidal magnetic particles, as estimated by Eq. 1 ^46^,

1 $${L}_{m}={({V}_{0}/N)}^{1/3}$$

where *V*_0_ is the initial volume of the sessile droplet and *N* is the total number of particles in the bulk of the droplet. In COMSOL, the magnetic field strength *H* is calculated by solving the Gauss’ law equation for magnetism,

2 $$-\nabla ({\mu }_{0}\nabla {V}_{m}-{\mu }_{0}M)=0$$

where $H=-\,\nabla {V}_{m}$; *M* is magnetization; *μ*_0_ is the vacuum permeability; and *V*_*m*_ is scalar magnetic potential. The particle motion is governed by Lagrangian equation (Eq. 3) that involves various forces such as drag force *F*_*D*_, magnetophoretic force *F*_*MG*_, Brownian force *F*_*b*_, gravitational force *F*_*g*_, and buoyancy force *F*_*bo*_,

3 $$m\frac{{d}^{2}s}{d{t}^{2}}={F}_{MG}+{F}_{D}+{F}_{b}+{F}_{g}+{F}_{bo}\,$$

where *m*, *s*, and *t* are the mass of the particle, the spatial coordinate of the particle, and time, respectively. The magnetophoretic force (*F*_*MG*_) is defined as:

4 $${F}_{MG}=2\pi {r}_{p}^{3}{\mu }_{0}{\mu }_{r,f}K\nabla {H}^{2}$$

where *r*_*p*_ is the radius of the particle; and *K* is defined as:

5 $$K=\frac{{\mu }_{r,p}-{\mu }_{r,f}}{{\mu }_{r,p}+2{\mu }_{r,f}}$$

where*μ*_*r*,*p*_ is the relative permeability of the ferromagnetic particles and *μ*_*r*,*f*_ is the relative permeability of the fluid. In COMSOL the drag force (*F*_*D*_) is expressed by Eq. 6,

6 $${F}_{D}=(\frac{1}{{\tau }_{p}}){m}_{p}(u-v)$$

where ${\tau }_{p}$ is the particle response time; *m*_*p*_ is the particle mass; *u* is the fluid velocity; and *v* is the velocity of the particle. The particle velocity response time for spherical particles in stokes flow is given by Eq. 7,

7 $${\tau }_{p}=\frac{{\rho }_{p}{d}_{p}^{2}}{18\mu }\,$$

where ${\rho }_{p}$ is the density of the particle, *d*_*p*_ is the particle diameter, and *μ* is the fluid viscosity. In this model, we assume the average particle size is 5 µm, and 30 nL droplet size as the simulation domain in which the particles are released. It is common for particles larger than 1 µm to be nearly unaffected by the Brownian motion, especially in highly viscous fluids such as terpineol oil. Furthermore, the gravitational and buoyancy forces, initially included in our simulations, were found to have negligible effects on the chaining time, vertical deposition velocity and time, indicating dominance of magnetophoretic and drag forces. Therefore, only the magnetophoretic force and the drag force are considered in this study.

On the other hand, estimating the characteristic time for the magnetic particles to chain (*t*_*ch*_) required only the particle tracking in the droplet. The terpineol oil is considered as the fluid in the domain. Two particles were released in the domain and treated as magnetic dipoles in a uniform magnetic field. The two particles can be treated as two magnetic dipoles, whose magnetic moments orient in the same direction as the external magnetic field. The induced magnetic dipoles in the applied magnetic field was calculated according to the specifications of the magnet used in our experiments. Figure S5 is the two-dimensional (2D) spatial representation of a magnetic dipole pair. The magnetic forces between the two dipoles are derived in both x- and y- directions as expressed in Eq. 8 and Eq. 9 ^47^,

8 $${F}_{x}=\frac{3{\mu }_{0}{m}_{1}{m}_{2}[1-5co{s}^{2}(\alpha )]\sin (\alpha )}{4\pi {r}^{4}}$$

9 $${F}_{y}=\frac{3{\mu }_{0}{m}_{1}{m}_{2}[3-5co{s}^{2}(\alpha )]\cos (\alpha )}{4\pi {r}^{4}}$$

where *m*_1_ and *m*_2_ are the two magnetic dipole moments, ${m}_{1}={m}_{2}=4\pi K{{r}_{p}}^{3}H$; *α* is the orientation angle of the dipoles with respect to the magnetic field; *r* is the dipole-diploe distance. The estimated characteristic times (*t*_*ch*_) and (*t*_*pz*_) were compared with the experimentally determined critical time for solvent imbibition (*t*_*im*_) to identify the conditions for chain formation and CRE suppression.

## Supplementary information

Supplementary Information

Supplementary Video 1

Supplementary Video 2

Supplementary Video 3

Supplementary Video 4

Supplementary Video 5

Supplementary Video 6

Supplementary Video 7
