# Supplementary material for: Inkjet Printing of Magnetic Particles Toward Anisotropic Magnetic Properties
Source: Sci Rep. 2019 Nov 7;9:16261. doi: 10.1038/s41598-019-52699-0 (PMC6838153; doi:10.1038/s41598-019-52699-0)
Supplement: Supplementary file 1 — Supplementary Information [file 41598_2019_52699_MOESM1_ESM.pdf]

Supporting Information

Inkjet Printing of Magnetic Particles Toward Anisotropic Magnetic Properties

Karam Nashwan Al-Milaji,[1] *Ravi L. Hadimani,*[2] *Shalabh Gupta*[3], *Vitalij K. Pecharsky*[3,4], *Hong Zhao*[1*]

[1] Virginia Commonwealth University, Department of Mechanical and Nuclear Engineering, BioTech One, 800 East Leigh Street, Richmond, VA, 23219, USA

[2] Virginia Commonwealth University, Department of Mechanical and Nuclear Engineering, 401 West Main Street, Richmond, VA, 23284, USA

[3] Ames Laboratory of the U.S. Department of Energy, Division of Materials Science and Engineering, Iowa State University, Ames, IA, 50011-2416, USA

[4] Department of Materials Science and Engineering, Iowa State University, Ames, IA, 50011-1096, USA

[*] To whom correspondences should be addressed:

Tel: 804-827-7025

Fax: 804-827-7030

hzhao2@vcu.edu

Video S1: Demonstration of the ink droplet imbibition into the photopaper. The droplet size is 30 nL. Particle concentration is 25 mg/mL.

Video S2: Demonstration of the dipole chaining process simulated in COMSOL. One dipole is placed at the center of the domain, whereas the second dipole is positioned at an orientation angle of 30° to the first one with respect to the y-axis (i.e., the direction of the magnetic field). The initial distance between the two diploes is selected as the mean particle distance (L_m), calculated at the particle concentration of 25 mg/mL.

Video S3 – S6: Demonstration of the dipole chaining process, with orientation angles of 0°, 45°, 60°, and 90°, respectively. The distance between the two diploes is the same as in Video S2.

Video S7: Demonstration of particle migration toward the substrate under the influence of the magnetophoretic force. The particles were released at a vertical distance (L_m) away from the substrate.

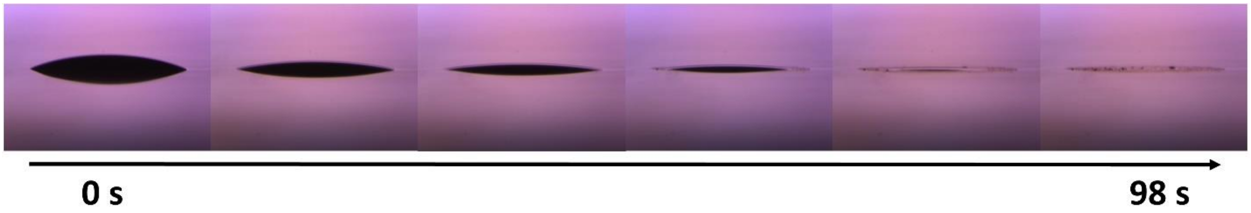

Figure S1: Illustration of the solvent imbibition process and the contact line pining. The droplet size is 30 nL. Particle concentration is 25 mg/mL.

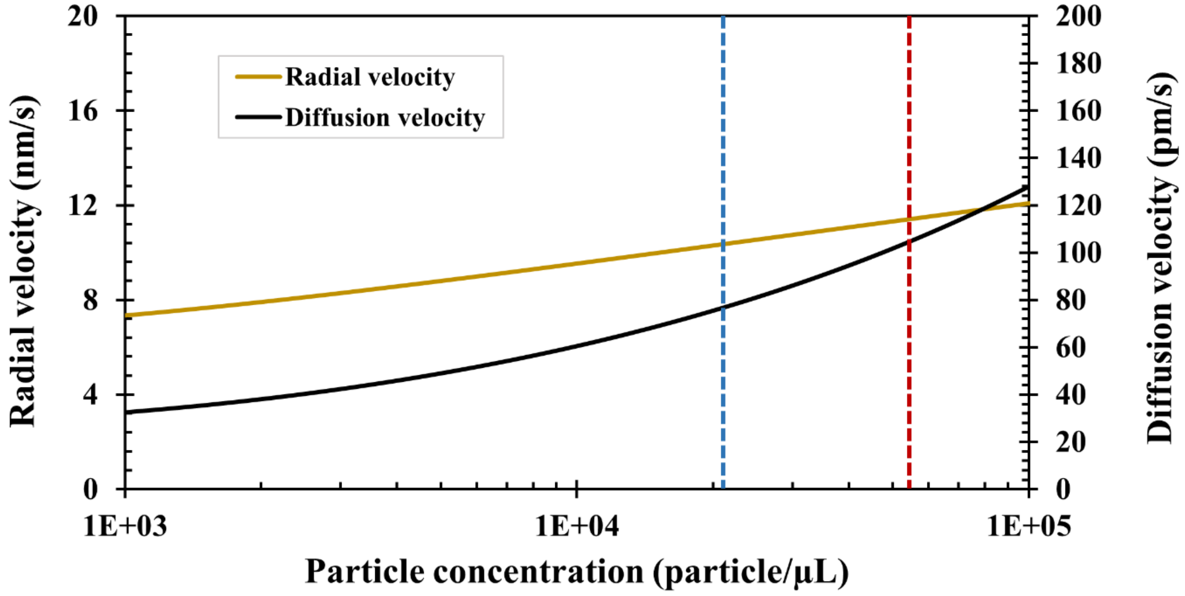

Figure S2: Evaporation-induced radial and diffusion velocities of the magnetic particles in terpineol oil. The mean particle size is 5 µm. The blue and red dash lines represent the particle concentrations of 10 mg/mL and 25 mg/mL, respectively. Please refer to ref. S1 for the calculation of radial and diffusion velocities in an evaporating sessile droplet.

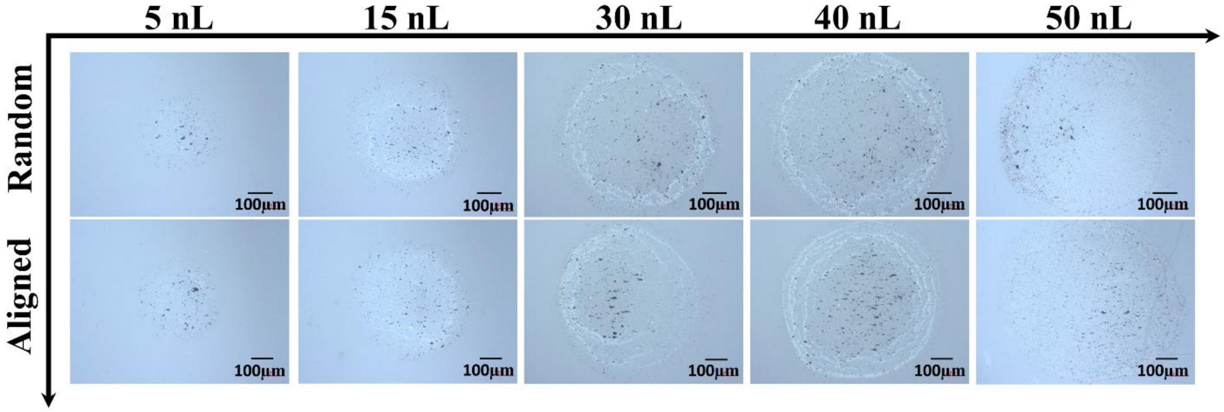

Figure S3: Optical microscope images of particle depositions of different droplet volumes, printed with 10 mg/mL particle concentration. “Random” refers to samples printed without applying external magnetic field and “Aligned” refers to samples with one-dimensional chains of particles when the magnetic field is applied during the printing process.

The particle depositions are somewhat inhomogeneous due to the following reasons: First, insufficient number of particles in the low concentration ink (10 mg/mL) results in depositions that are incompletely filled with particles. Second, it is nearly impossible to fully flatten the substrate, and locally it has minor but varying curvatures. These local differences force variable downward flows, especially when droplets are larger than 30 nL, resulting in inhomogeneous particle depositions. Third, some bright successive rings inside the particle depositions, especially for the 10 mg/mL particle concentration, further contribute to the inhomogeneity of the particle depositions.

The observed inhomogeneities do not affect the main conclusion that the CRE can be suppressed and particle chains formed with external magnetic field. Without magnetic field, the CRE becomes noticeable at droplet volumes greater than 40 nL for the 10 mg/mL particle concentration. While with magnetic field, the CRE has been suppressed especially for larger droplets.

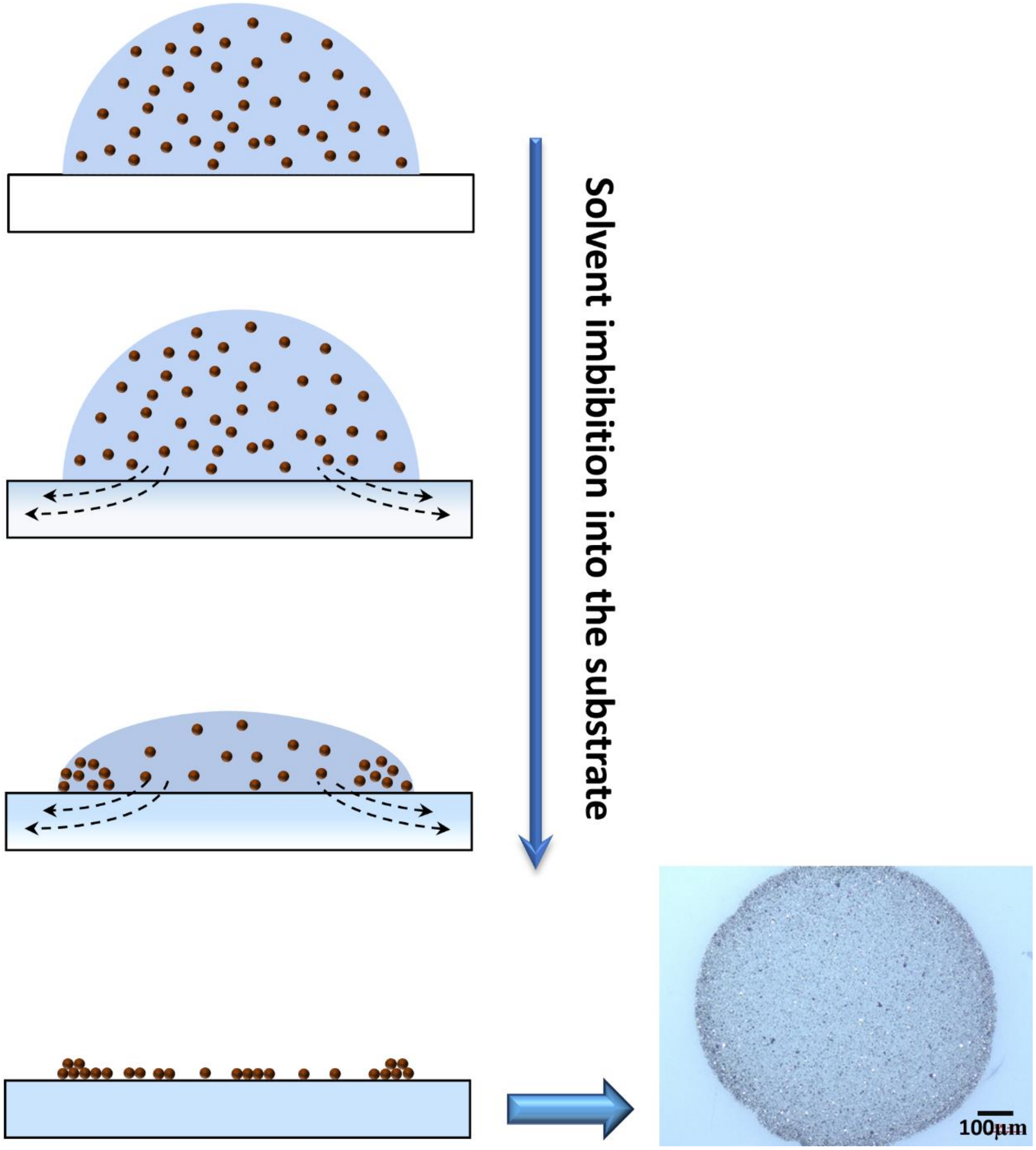

Figure S4: Illustration of solvent infiltration mechanism into the photopaper for droplets bigger than 30 nL. Dashed arrows indicate the solvent infiltration. When no magnetic field is applied, the particles follow the direction of the flow as the solvent seeps into the porous substrate beyond the three-phase contact line, resulting in a ring-like particle deposition.

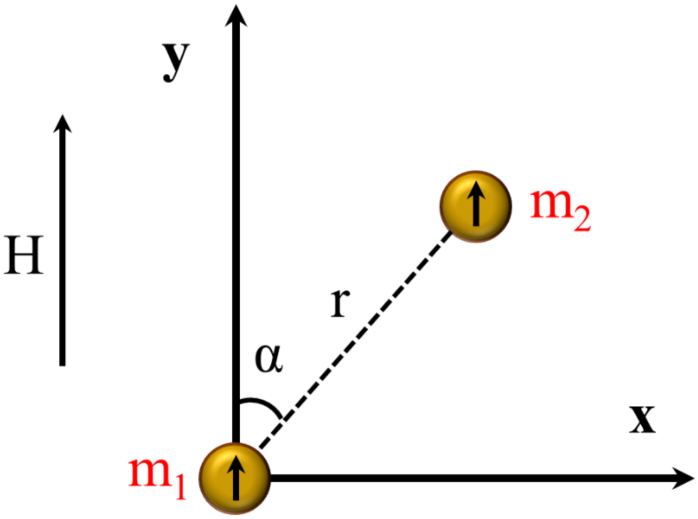

Figure S5: Spatial representation of two magnetic dipoles in a uniform magnetic field.

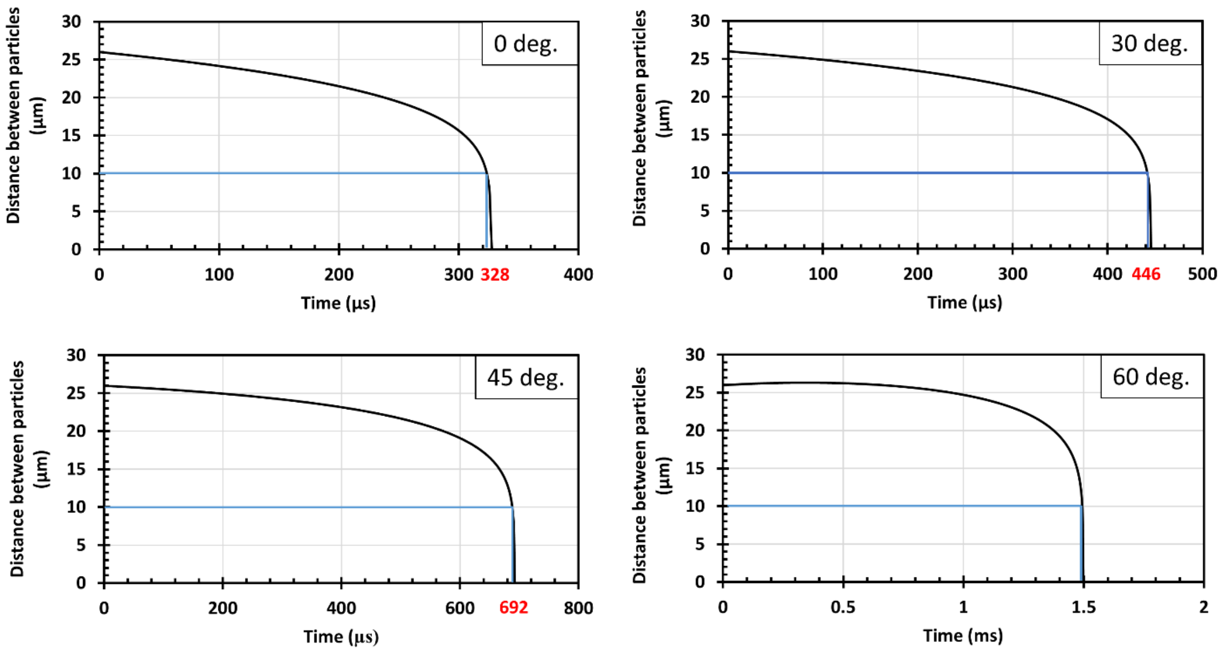

Figure S6: Distance between the two dipoles during the chaining process. The characteristic time for the two dipoles to collide is obtained when this distance reaches 10 μm, which is two times of the particle radius. The characteristic times for chaining t_{ch} are 328 μs, 446 μs, 692 μs, and 1.5 ms, for the orientation angles of 0°, 30°, 45°, and 60°, respectively.

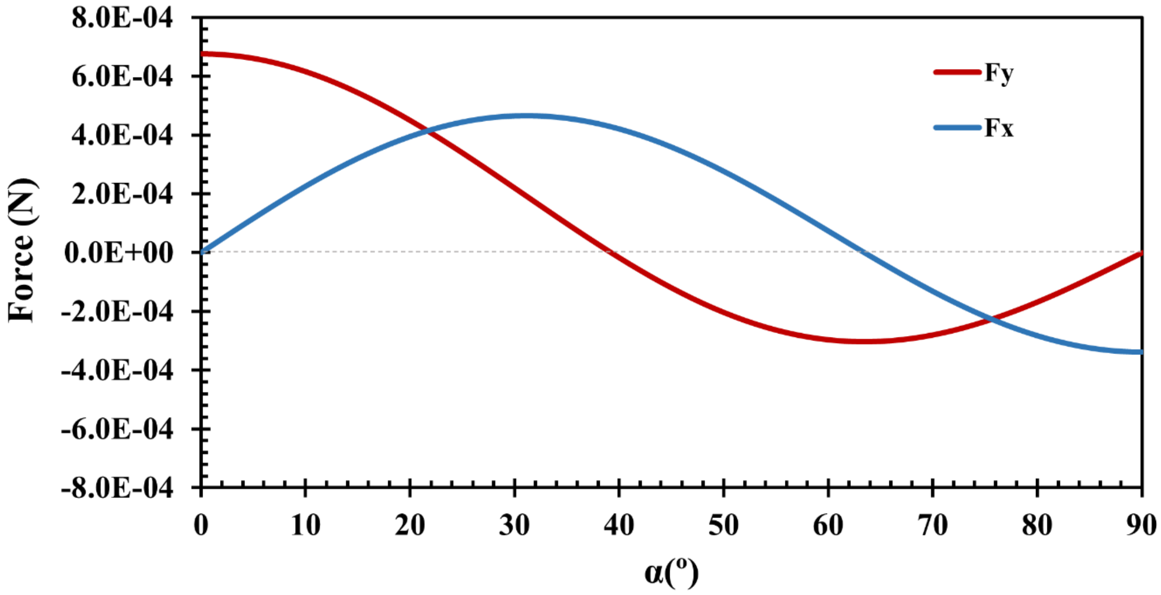

Figure S7: Interaction forces acting between the two dipoles in the simulation domain. Positive forces represent attractive forces between the two particle dipoles; and the negative forces are repulsive forces.

As can be seen from Figure S7, the interaction force on the magnetic dipole m_2 varies with respect to the orientation angle. When the orientation angle is set to zero, i.e., the two dipoles are arranged along the direction of the applied magnetic field, the interaction force in the y-direction is strongest in attraction. It then decreases from its maximum to zero at α = 39° and becomes repulsive between α = 39° to α = 64°, followed by a decline to zero at α = 90°. On the other hand, the interactions force in the x-direction is zero at α = 0°. Then it increases to its maximum at α = 32°, followed by a decrease to zero at α = 63°. Finally, it become most repulsive at α = 90°.

When both dipoles are placed on the x-axis ($\alpha = 90°$), a repulsion force is developed to separate the dipoles apart. On the contrary, the dipoles attract the most when the angle ($\alpha = 0°$) as shown in video S3. In this case, the particle velocity continuously increases until the dipoles collide. For any orientation angles in between, the dipoles follow elliptical trajectories until they collide and align themselves with the y-axis, i.e., the direction of the magnetic field. The chaining characteristic time (t_{ch}) varies in these cases, where longer particle chaining time is observed when the orientation angle (α) approaches 90°. This could be attributed to development of the successive, mutual repulsion or attraction forces obtained at different dipole orientations.

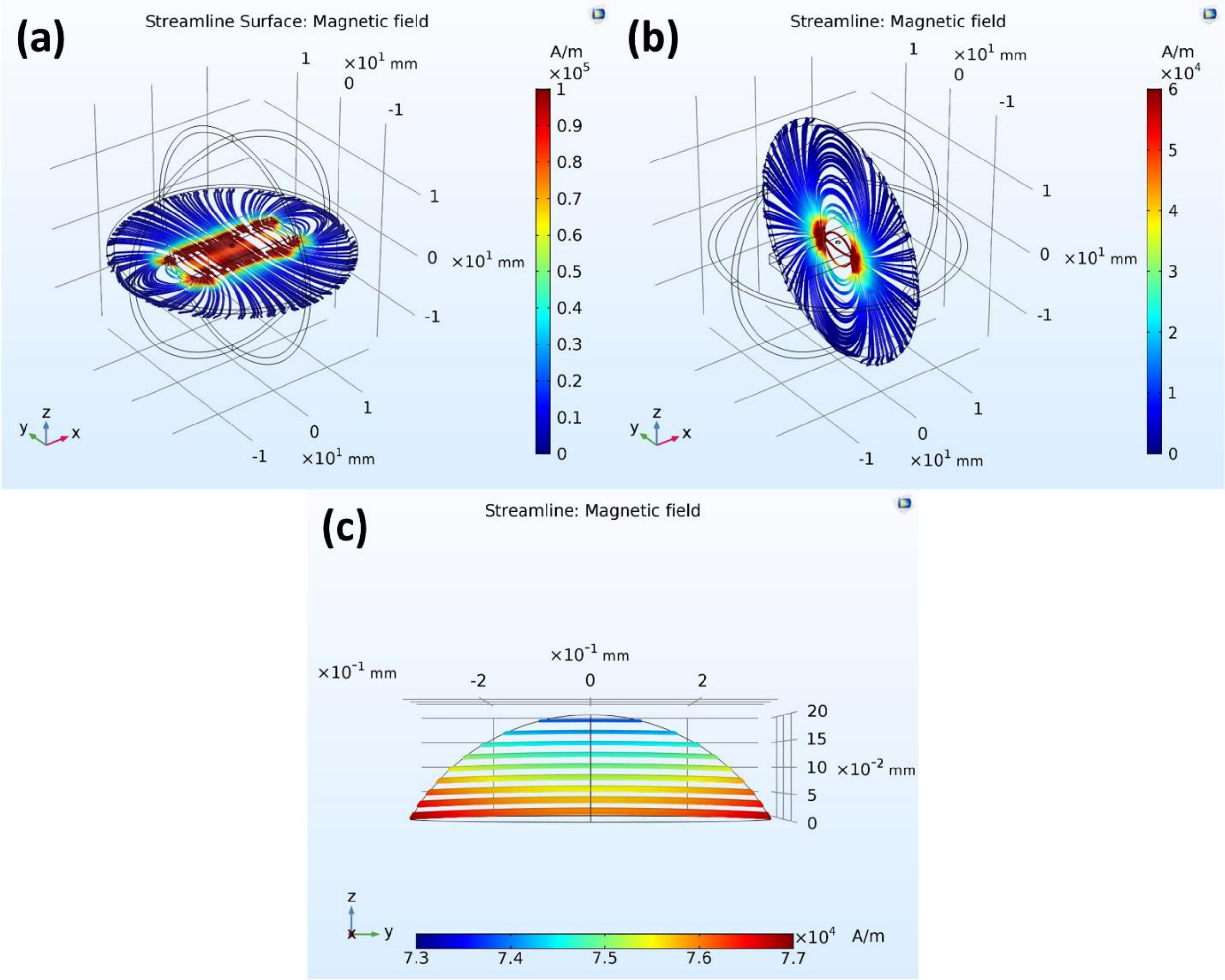

Figure S8: (a) and (b) Magnetic field simulation, which illustrates the magnetic field in XY and YZ planes. One droplet was placed on the top center of the permanent magnet. (c) Illustration of the magnetic field gradient inside the sessile droplet.

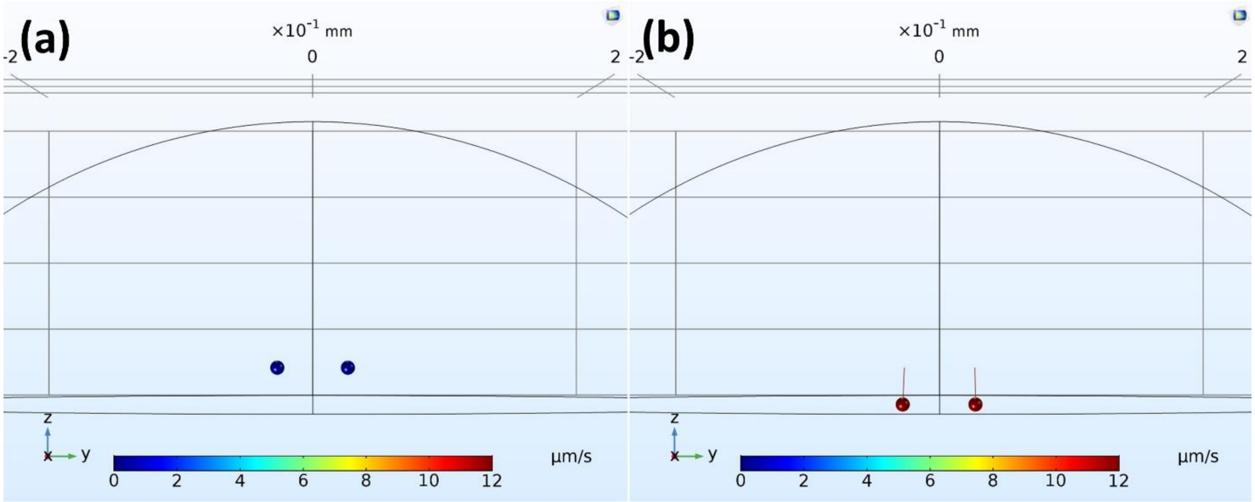

Figure S9: (a) Two particles released in the simulation domain with an initial velocity of zero; (b) particle migration in the z-direction toward the substrate under the influence of the magnetophoretic force and drag force. The particles were positioned at a vertical distance (L_m) away from the substrate, to calculate the characteristic time (t_{pz}) for the magnetic particles to reach the substrate.

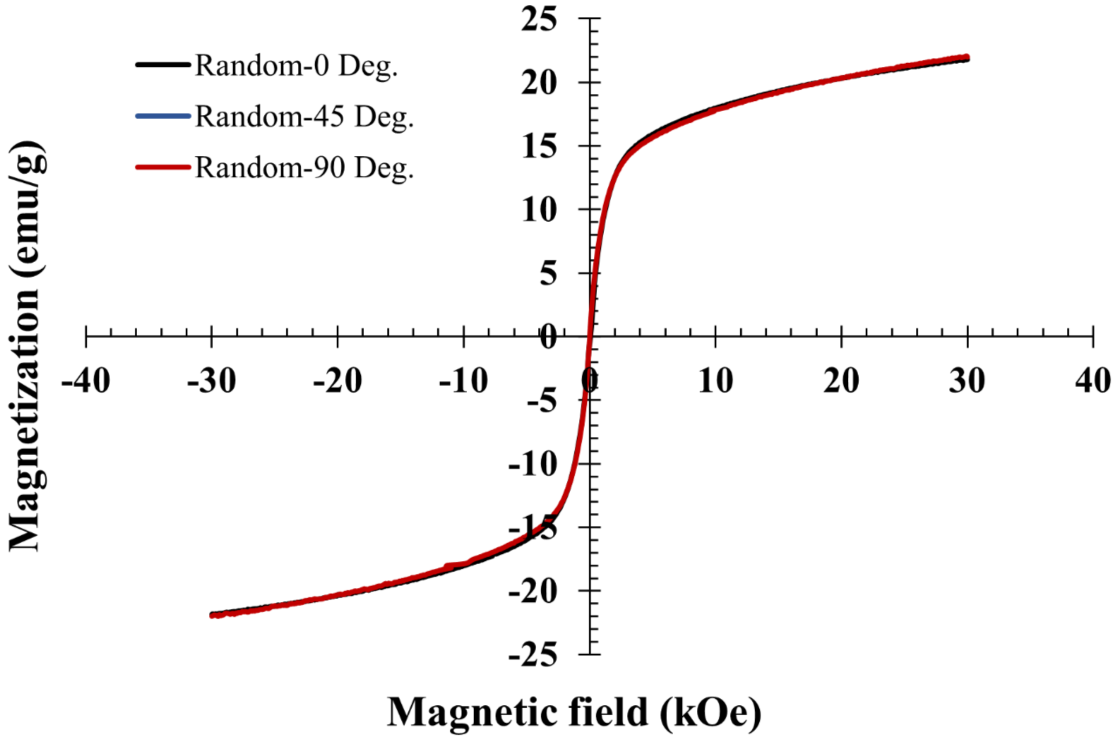

Figure S10: Magnetization curves of the printed "Random" films at different orientation angles with respect to the measuring magnetic field. "Random" refers to films printed without external magnetic field and with random particle deposition.

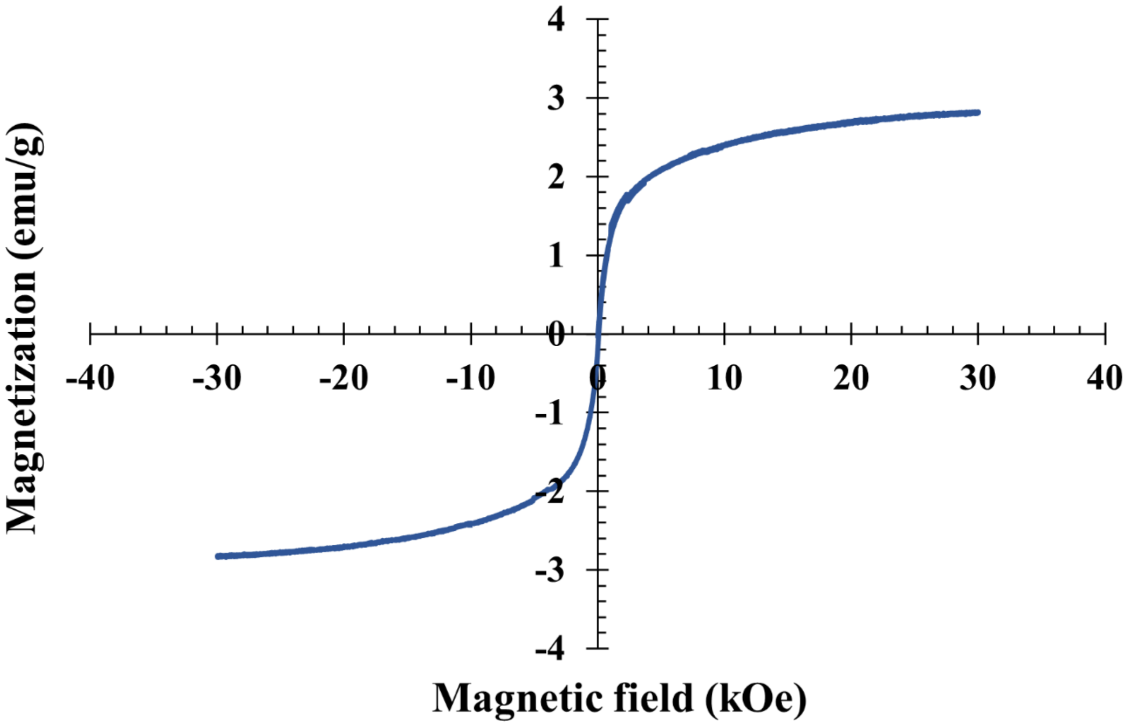

Figure S11: Magnetization curve of the photopaper.

The photopaper substrate has a weak but measurable magnetic response as shown in Figure S11. It is likely due to the coating materials on the photopaper.

References

S1. Al-Milaji, K. N. & Zhao, H. Probing the Colloidal Particle Dynamics in Drying Sessile Droplets. *Langmuir* **35**, 2209-2220 (2019).
